# A health worker knowledge, attitudes and practices survey of SARS-CoV-2 infection prevention and control in South Africa

**DOI:** 10.1186/s12879-021-05812-6

**Published:** 2021-02-01

**Authors:** Saiendhra Vasudevan Moodley, Muzimkhulu Zungu, Molebogeng Malotle, Kuku Voyi, Nico Claassen, Jonathan Ramodike, Nkululeko Thunzi, Nosimilo Mlangeni

**Affiliations:** 1 School of Health Systems and Public Health, University of Pretoria, Pretoria, South Africa; 2 National Institute for Occupational Health, A division of the National Health Laboratory Service, Johannesburg, South Africa; 3 Department of Community Health, Sefako Makgatho Health Sciences University, Ga-Rankuwa, South Africa

**Keywords:** SARS-CoV-2, COVID-19, Health workers, Infection prevention, Knowledge, Attitudes, Perceptions, Practices

## Abstract

**Background:**

Health workers are crucial to the successful implementation of infection prevention and control strategies to limit the transmission of SARS-CoV-2 at healthcare facilities. The aim of our study was to determine SARS-CoV-2 infection prevention and control knowledge and attitudes of frontline health workers in four provinces of South Africa as well as explore some elements of health worker and health facility infection prevention and control practices.

**Methods:**

A cross-sectional study design was utilised. The study population comprised both clinical and non-clinical staff working in casualty departments, outpatient departments, and entrance points of health facilities. A structured self-administered questionnaire was developed using the World Health Organization guidance as the basis for the knowledge questions. COVID-19 protocols were observed during data collection.

**Results:**

A total of 286 health workers from 47 health facilities at different levels of care participated in the survey. The mean score on the 10 knowledge items was 6.3 (SD = 1.6). Approximately two-thirds of participants (67.4%) answered six or more questions correctly while less than a quarter of all participants (24.1%) managed to score eight or more. A knowledge score of 8 or more was significantly associated with occupational category (being either a medical doctor or nurse), age (< 40 years) and level of hospital (tertiary level). Only half of participants (50.7%) felt adequately prepared to deal with patients with COVD-19 at the time of the survey. The health workers displaying attitudes that would put themselves or others at risk were in the minority. Only 55.6% of participants had received infection prevention and control training. Some participants indicated they did not have access to medical masks (11.8%) and gloves (9.9%) in their departments.

**Conclusions:**

The attitudes of participants reflected a willingness to engage in appropriate SARS-CoV-2 infection prevention and control practices as well as a commitment to be involved in COVID-19 patient care. Ensuring adequate infection prevention and control training for all staff and universal access to appropriate PPE were identified as key areas that needed to be addressed. Interim and final reports which identified key shortcomings that needed to be addressed were provided to the relevant provincial departments of health.

## Background

Coronavirus disease 19 (COVID-19) is a communicable disease caused by a novel coronavirus designated SARS-CoV-2 (severe acute respiratory syndrome coronavirus 2) [1]. Evidence indicates that SARS-CoV-2 has a zoonotic source [2]. This animal coronavirus spill over to humans is the third one to be documented in the past twenty years [1]. Human-to-human transmission of SARS-CoV-2 occurs through close contact with an infected individual via respiratory droplets, direct contact with infected individuals through infected secretions, or indirect contact through touching contaminated surfaces [3]. There is also increasing evidence for the airborne spread of SARS-CoV-2 with enclosed environments with poor ventilation of particular concern [4–6].

Africa’s first recorded case of COVID-19 was in Egypt on 14 February 2020 [7]. South Africa reported its first case of COVID-19 on 5 March 2020 in a 38-year old male who had returned to South Africa after a trip to Italy [8]. South Africa’s daily COVID-19 case numbers started increasing significantly from mid-March 2020 reaching the highest daily recorded number of cases in its first wave of 13,944 on 25 June 2020 [9]. By 31 October 2020, South Africa had recorded 723,682 confirmed COVID-19 cases and 19,230 COVID-19 deaths which represented over 40% of all confirmed COVID-19 cases and approximately 45% of all COVID-19 deaths in Africa at the time [9]. However, excess deaths data for South Africa suggest that the number of COVID-19 deaths in South Africa was substantially higher [7].

A study conducted in the United Kingdom as well as in the United States found that frontline healthcare workers are more likely to test positive for COVID-19 than the general community [10]. Inpatient settings appeared to pose the most risk followed by nursing homes, and outpatient hospital clinics [10]. There has been a significant number of COVID-19 deaths among health workers globally. It was reported in early September that the number of COVID-19 deaths among health workers worldwide was at least 7000 [11]. The countries that were estimated to have the most deaths of health workers were Mexico (1320), the United States of America (1077) and the United Kingdom (649) [11]. South Africa with 240 estimated deaths at the time was seventh on the list [11].

Infection prevention and control at healthcare facilities is critical in limiting transmission of SARS-CoV-2 to health workers and patients. The World Health Organization (WHO) has identified a number of strategies to reduce the risk of transmission of SARS-CoV-2 in health settings including isolation of suspected cases, the application of standard precautions to all patients, and the implementation of additional precautions for suspected COVID-19 cases [12]. Administrative controls should be implemented and engineering and environmental controls should be utilised to limit transmission in health facilities [12]. This is echoed by South African guidelines [13]. Health workers are crucial in successful implementation of infection prevention and control strategies. The aim of our study was, therefore, to determine SARS-CoV-2 infection prevention and control knowledge and attitudes of frontline health workers in four provinces of South Africa. In addition, we explored some elements of health worker and health facility infection prevention and control practices.

## Methods

### Study design

A cross-sectional study design was utilised with both descriptive and analytical components. The knowledge, attitudes and practices (KAP) survey was part of a rapid appraisal of the COVID-19 occupational health and safety response in four provinces in South Africa.

### Study setting

The study was conducted in public sector (government-run) facilities in four of the nine provinces of South Africa viz. Gauteng Province, Limpopo Province, Mpumalanga Province, and North-West Province. The public sector facilities comprised district, regional, tertiary, central, and specialised psychiatric hospitals as well as community health centres.

### Study population and sampling

The study population comprised frontline health workers within the four provinces that are based in casualty (accident and emergency) departments, outpatient departments, and entrance points of health facilities. These are likely to be the initial points of contact between undiagnosed COVID-19 cases and health facility staff thereby placing workers stationed in these areas at risk of COVID-19 transmission. This included both clinical and non-clinical staff. The health facilities from which participants were drawn were conveniently sampled. At each facility, the target was to invite at least one health worker from each of six categories of staff to participate viz. medical doctors, nurses, cleaners, porters, security personnel and clerks. Clinical associates (similar cadre to physician assistants) were also invited to participate but are not employed at all facilities. Health workers were selected at each facility based on their availability at the time of the rapid appraisal. This was done to minimise disruption to normal service delivery.

### Measurement tool and data collection

A structured questionnaire with closed-ended questions in four sections (socio-demographic characteristics, knowledge items, attitude/perception items and practice items) was utilised for data collection which took place over a two-month period commencing mid-April 2020. The knowledge items of the KAP survey were based on the WHO interim guidance of 25 January 2020 [12]. The WHO interim guidance released on 19 March 2020 did not reflect any changes with respect to the knowledge items included in our questionnaire [14]. For the knowledge items, participants were provided with a statement and had to indicate if they agreed, disagreed or were unsure. The attitude and perception items used a five-point Likert response scale (strongly disagree; disagree; unsure; agree; strongly agree). The practice items measured both individual and facility practices with the WHO guidance being used as the basis for most of the items [12]. The responses from which participants could select for the practice items were ‘yes’, ‘no’, ‘not sure’, and ‘not applicable’. The ‘not applicable’ option was provided as not all practice items were applicable to all categories of health workers and ‘not applicable’ responses were not included in the data analysis. The questionnaires were self-administered with a researcher or trained research assistant being in the vicinity to address any queries. Physical distancing, wearing of medical masks and hygiene measures were employed to reduce the risk of transmission of SARS-CoV-2 during the data collection process.

### Data management and analysis

Data entry was done on Microsoft Excel and imported into STATA version 16 (v15 (Statacorp; http://www.stata.com) for analyses. For the knowledge items, ‘unsure’ and non-responses were treated as being incorrect and were included in the denominator when calculating the proportion of correct responses. The number of questions answered correctly were added to give a score out of 10. Participants were classified as having adequate knowledge if they answered six or more questions correctly and good knowledge if they answered 8 or more questions correctly. Logistic regression was utilised to determine factors associated with a good knowledge score. Socio-demographic and employment variables with a *p*-value less than 0.25 on univariate logistic regression were included in the initial multivariate logistic regression model. The final multivariate model was developed by dropping variables from the initial model using a backwards stepwise approach. For each attitude/perception item, the proportion of participants selecting each Likert-scale option was calculated. Proportions were also calculated for each of the practice items.

## Results

### Socio-demographic characteristics of participants

A total of 286 health workers from 47 health facilities participated in the survey. The health facilities comprised 4 community health centres, 22 district hospitals, 11 regional hospitals, 7 tertiary hospitals, a central hospital, and 2 specialised psychiatric hospitals. The socio-demographic and employment characteristics of the participants are displayed in Table 1. The majority of participants were aged between 30 and 49 years (62.9%) and the median age was 39 years. There was substantially more female (59.6%) than male (40.4%) participants. A large proportion of participants (46.6%) did not possess a diploma or degree. Except for clinical associates (1.8%), the different occupational categories of frontline staff were well represented. The ‘other’ category included various occupational categories such as allied health professionals, pharmacists, and pharmacy assistants. As would be expected based on the facilities selected, the largest proportion of participants (45.1%) worked at district hospitals.

**Table 1:** Socio-demographic and employment characteristics of participants

Characteristic (N)	Categories	n	Percentage (%)
Age (*N* = 264)	18–29 years	45	17.0
	30–39 years	92	34.8
	40–49 years	74	28.0
	50–59 years	46	17.4
	≥ 60 years	7	2.7
Gender (*N* = 277)	Female	165	59.6
	Male	112	40.4
Education – highest level completed (N = 264)	Primary school	6	2.3
	Secondary school	117	44.3
	Diploma	68	25.8
	Undergraduate degree	41	15.5
	Postgraduate degree	32	12.1
Occupation (*N* = 279)	Cleaner	40	14.3
	Clerk	44	15.8
	Clinical associate	5	1.8
	Medical doctor	42	15.1
	Nurse	49	17.6
	Porter	32	11.5
	Security	42	15.1
	Other	25	9.0
Province of employment (*N* = 286)	Gauteng	57	19.9
	Limpopo	56	19.6
	Mpumalanga	78	27.3
	North West	95	33.2
Type of facility where employed (N = 286)	Community health centre	21	7.3
	District hospital	129	45.1
	Regional hospital	67	23.4
	Tertiary hospital	46	16.1
	Central hospital	7	2.5
	Specialised psychiatric hospital	16	5.6
Years of experience (*N* = 206)	0–9 years	124	60.2
	10–19 years	50	24.3
	20–29 years	15	7.3
	≥ 30 years	17	8.3

### Health worker knowledge

The proportions of health workers who responded correctly to each of the knowledge items are shown in Table 2. More than 90% of participants provided correct answers on each of the two items related to the mechanism of transmission of SARS-CoV-2. Just over half of participants (52.1%) knew that droplet and contact precautions were required for both confirmed cases of COVID-19 and for suspected cases. The lowest proportion of correct responses (6.6%) was obtained for one of the questions related to the appropriate personal protective equipment (PPE) to be used in the routine care of COVID-19 patients. In total, there were three items where the proportion of correct responses fell below 50%. Medical doctors attained the highest or joint-highest proportion of correct responses on eight of the ten items.

**Table 2:** SARS-CoV-2 infection prevention and control knowledge items by occupational category

	Cleaner (*N* = 40)	Clerk (*N* = 44)	Doctor (*N* = 42)	Nurse (*N* = 49)	Porter (*N* = 32)	Security (N = 42)	Other (*N* = 30*)	All (*N* = 286^#^)
**Knowledge item correct responses n (%)** *(Correct responses in parentheses)*								
COVID-19 is transmitted during close contact through respiratory droplets. (Agree)	32 (80.0)	42 (95.5)	42 (100.0)	47 (95.9)	29 (90.6)	40 (95.2)	30 (100.0)	267 (93.4)
A person can become infected with COVID-19 by touching surfaces where COVID-19 droplets have landed and then touching their face. (Agree)	39 (97.5)	44 (100.0)	42 (100.0)	49 (100.0)	31 (96.9)	42 (100.0)	29 (96.7)	282 (98.6)
Droplet and contact precautions are required for confirmed cases of COVID-19 but not for suspected cases. (Disagree)	16 (40.0)	18 (40.9)	37 (88.1)	28 (57.1)	15 (46.9)	12 (28.6)	21 (70.0)	149 (52.1)
Airborne transmission of COVID-19 droplets is usually of distances of 3 m or more. (Disagree)	18 (45.0)	23 (52.3)	36 (85.7)	35 (71.4)	17 (53.1)	23 (54.8)	20 (66.7)	175 (61.2)
Hand hygiene with alcohol-based hand rubs is always preferred over soap and water. (Disagree)	13 (32.5)	17 (38.6)	28 (66.7)	27 (55.1)	11 (34.4)	5 (11.9)	13 (43.3)	115 (40.2)
Boots, coveralls and aprons are required in the routine care of COVID-19 patients. (Disagree)	3 (7.5)	3 (6.8)	4 (9.5)	3 (6.1)	2 (6.3)	4 (9.5)	0 (0.0)	19 (6.6)
Medical masks should be used during the routine care of COVID-19 patients. (Agree)	28 (70.0)	38 (86.4)	33 (78.6)	39 (79.6)	26 (81.3)	37 (88.1)	25 (83.3)	232 (81.1)
N95 respirators should be used for procedures in COVID-19 patients that are aerosol-generating. (Agree)	31 (77.5)	29 (65.9)	40 (95.2)	44 (89.8)	30 (93.8)	34 (81.0)	22 (73.3)	235 (82.2)
Healthcare workers should use gloves during the routine care of COVID-19 patients. (Agree)	36 (90.0)	41 (93.1)	41 (97.6)	49 (100.0)	31 (96.9)	39 (92.9)	30 (100.0)	272 (95.1)
Patients with suspected COVID-19 infection should be given N95 respirators to prevent transmission to healthcare workers. (Disagree)	6 (15.0)	4 (9.1)	27 (64.3)	21 (42.9)	3 (9.4)	2 (4.8)	3 (10.0)	66 (23.1)
**Summary statistics for 10 knowledge items**								
Mean (SD)	5.6 (1.5)	5.9 (1.1)	7.9 (1.2)	7.0 (1.4)	6.1 (1.5)	5.7 (1.2)	6.4 (1.3)	6.3 (1.6)
Median	5	6	8	7	6	6	7	6
Number (%) of participants with score ≥ 6	17 (42.5)	24 (54.6)	40 (95.2)	42 (85.7)	23 (71.9)	22 (52.4)	20 (66.7)	188 (67.4)
Number (%) of participants with score ≥ 8	4 (10.0)	4 (9.1)	27 (64.3)	18 (36.7)	5 (15.6)	2 (4.8)	8 (26.7)	69 (24.1)

* includes 5 clinical associates ^#^includes 7 health workers who did not provide their occupational category

The mean score on the 10 knowledge items was 6.3 (SD = 1.6) (Table 2). Medical doctors had the highest mean score of 7.9 (SD = 1.2) followed by nurses with a mean score of 7.0 (SD = 1.4). The difference between doctors and nurses was statistically significant (*p* = 0.002). Cleaners, clerks and security personnel all had mean scores below 6. Approximately two-thirds of participants (67.4%) answered six or more questions correctly while less than a quarter of all participants (24.1%) managed to score eight or more. The majority of participants in all but one of the health worker categories answered six or more of the questions correctly. With the exception of medical doctors, the majority of participants in each of the health worker categories failed to achieve a minimum of eight correct responses.

The final multivariate model for factors associated with a knowledge score of ≥8 is shown in Table 3. The initial model consisted of sociodemographic characteristics with *p* < 0.25 following univariate logistic regression viz. age, education, occupation, years of experience and type of facility where employed. Health workers aged 40 years and older had lower odds of getting 8 or more of the knowledge items correct. Medical doctors and nurses had higher odds of scoring 8 or more as did health workers employed at tertiary hospitals.

**Table 3:** Factors associated with knowledge score of ≥8 (final multivariate model) (*N* = 261)

Characteristic	Odds Ratio	95% Confidence Interval	p-value
**Age**			
18–39 years	1.00		
≥40 years	0.37	0.18–0.75	0.006
**Occupation**			
Cleaner	1.00		
Clerk	0.97	0.21–4.55	0.968
Medical doctor	16.43	4.43–61.0	< 0.001
Nurse	6.42	1.77–23.27	0.005
Porter	1.77	0.39–7.97	0.457
Security	0.36	0.06–2.25	0.273
Other	3.53	0.81–15.31	0.093
**Type of facility where employed**			
Community health centre	1.00		
District hospital	1.17	0.27–5.17	0.834
Regional hospital	1.03	0.20–5.11	0.970
Tertiary hospital	6.01	1.19–30.48	0.030
Central hospital	5.44	0.50–59.56	0.165
Specialised psychiatric hospital	2.23	0.35–14.08	0.395

### Health worker attitudes and perceptions

Only half of participants (50.7%) felt adequately prepared to deal with patients with COVD-19 at the time of the survey (see Table 4). A majority of participants (86.1%) indicated they would wear the required PPE even if it were uncomfortable. Almost two-thirds of participants (65.1%) strongly disagreed with the statement that they would still treat a COVID-19 patient even if the required PPE was not available with a further 21.7% disagreeing with the statement. A substantial proportion of participants (46.8%) felt they could be infected with COVID-19 at their facility regardless of the precautions they take. One-tenth of participants (10.0%) indicated they would continue to report for duty even if they had symptoms suggestive of COVID-19. More than 5% of participants (6.4%) indicated they would resign to avoid contact with COVID-19 patients.

**Table 4:** SARS-CoV-2 infection prevention and control attitudes/perceptions

Statement	Strongly disagree n (%)	Disagree n (%)	Unsure n (%)	Agree n (%)	Strongly agree n (%)
I am adequately prepared to deal with patients with COVID-19. (*N* = 278)	45 (16.2)	40 (14.4)	52 (18.7)	106 (38.1)	35 (12.6)
I would wear the required personal protective equipment even if it is uncomfortable. (*N* = 280)	22 (7.9)	11 (3.9)	6 (2.1)	118 (42.1)	123 (43.9)
I would feel safer using a respirator rather than a medical mask when dealing with a patient with COVID-19. (*N* = 279)	28 (10.0)	42 (15.1)	28 (10.0)	85 (30.5)	96 (34.4)
I feel safer using alcohol-based hand rubs than washing my hands with soap and water. (*N* = 283)	49 (17.3)	62 (21.9)	13 (4.6)	101 (35.7)	58 (20.5)
I would still treat a COVID-19 patient even if the required personal protective equipment was not available. (*N* = 272)	177 (65.1)	59 (21.7)	12 (4.4)	16 (5.9)	8 (2.9)
I feel that I could be infected with COVID-19 at my facility regardless of the precautions I take. (*N* = 278)	54 (19.4)	55 (19.8)	39 (14.0)	85 (30.6)	45 (16.2)
I will continue to report for duty even if I get symptoms suggestive of COVID-19. (*N* = 279)	157 (56.3)	83 (29.8)	11 (3.9)	17 (6.1)	11 (3.9)
I am not too concerned about getting a severe COVID-19 infection as I am still relatively young. (*N* = 275)	144 (52.4)	77 (28.0)	18 (6.6)	23 (8.4)	13 (4.7)
I would stay away from work in order to avoid contact with COVID-19 patients. (*N* = 280)	108 (38.6)	109 (38.9)	17 (6.1)	20 (7.1)	26 (9.3)
I would resign from my job in order to avoid contact with COVID-19 patients. (N = 280)	163 (58.2)	92 (32.9)	7 (2.5)	5 (1.8)	13 (4.6)

### Health facility and health worker practices

Participant responses to questions related to their own practices as well as that of their health facility are shown in Table 5. Just over half of participants (51.3%) confirmed they had undergone occupational health and safety training. Slightly more than half of participants indicated they had received infection prevention and control training (55.6%) and training on the correct use of PPE (56.2%). Less than 90% of participants indicated they had access to medical masks (85.7%) and gloves (88.7%) with approximately half of participants (51.0%) indicating they had access to respirators in their department. The vast majority of participants indicated they practiced hand hygiene after touching a patient (97.7%) and after touching a patient’s surroundings (90.3%).

**Table 5:** Infection prevention and control practices

	Yes n (%)	No n (%)	Unsure n (%)
Participant received occupational health and safety training (*N* = 275)	141 (51.3)	126 (45.8)	8 (2.9)
Participant received infection prevention and control training (*N* = 275)	153 (55.6)	119 (43.3)	3 (1.1)
Participant received training on correct use of personal protective equipment (*N* = 274)	154 (56.2)	114 (41.6)	6 (2.2)
Infection prevention guidelines on COVID-19 available in participant’s department (*N* = 265)	193 (72.8)	48 (18.1)	24 (9.1)
Infection prevention posters on COVID-19 available in participant’s department (*N* = 271)	231 (85.3)	29 (10.7)	11 (4.1)
Access to medical masks in participant’s department (*N* = 272)	233 (85.7)	32 (11.8)	7 (2.6)
Access to respirators in participant’s department (*N* = 261)	133 (51.0)	110 (42.1)	18 (6.9)
Access to gloves in participant’s department (N = 274)	243 (88.7)	27 (9.9)	4 (1.5)
Access to soap and water in participant’s department (N = 271)	246 (90.8)	19 (7.0)	6 (2.2)
Access to alcohol-based hand rubs in participant’s department (*N* = 268)	244 (91.0)	20 (7.4)	4 (1.5)
Participant always practises hand hygiene (soap and water or alcohol-based hand rubs after touching a patient (N = 264)	258 (97.7)	5 (1.9)	1 (0.4)
Participant always practises hand hygiene (soap and water or alcohol-based hand rubs) after touching a patient’s surroundings (*N* = 269)	243 (90.3)	18 (6.7)	8 (3.0)
Participant cleans and disinfect equipment that is usually used for multiple patients (e.g. stethoscopes) prior to it being used on each new patient (*N* = 237)	195 (82.3)	24 (10.1)	18 (7.6)

## Discussion

Our study examined the knowledge, attitudes and practices of frontline health workers across occupational categories. We included both clinical and non-clinical personnel. The expectation was that knowledge of SARS-CoV-2 infection prevention and control would differ by occupational category and we, therefore, stratified our analysis of the knowledge items by occupation. When comparing mean scores, clinical personnel (doctors and nurses) fared better on their overall knowledge than non-clinical personnel. This was confirmed by our multivariate model with doctors and nurses having significantly higher odds of obtaining eight or more knowledge items correct. Doctors fared significantly better than nurses in our study with respect to mean knowledge scores. A study in Greece found a larger proportion of physicians received high scores on their knowledge of SARS-CoV-2 preventative practices in comparison to nurses [15]. A COVID-19 KAP study of healthcare workers in China found similar knowledge scores among doctors and nurses [16] while a COVID-KAP study among clinical personnel in Uganda found no significant difference in the level of COVID-19 knowledge of the different clinical cadres [17]. A survey of generic infection prevention in Ethiopia found physicians were significantly less knowledgeable with respect to infection prevention than nurses [18].

In addition to occupational category, we found that a good knowledge score was also significantly associated with age and type of facility where employed. Older health workers had lower odds of achieving a good knowledge score than younger health workers. This finding corresponds to the finding of Olum et al. [17] who used a similar cut off of 80% for knowledge items and reported significantly lower odds of achieving this in their participants older than 40 years. Two other COVID-19 KAP studies in health workers that have used knowledge scores have not shown a relationship between knowledge scores and age [15, 19]. Olum et al. [17] speculate that the higher knowledge scores seen in younger health workers may be related to their utilisation of a diversity of information sources. Infection prevention knowledge has been linked to years of experience but we did not find that to be a statistically significant association in our study [18]. In our study, health workers employed at tertiary hospitals had higher odds of achieving a good knowledge score. Tertiary hospitals in South Africa provide specialist, subspecialist and intensive care services with many involved in training of health professionals [20]. The higher knowledge scores obtained by health workers at tertiary hospitals may reflect the greater focus on infection prevention and control at this level. Based on a similar argument, one would expect health workers from central hospitals to also perform well but we are unable to draw any conclusions on this as there was only one central hospital in our sample.

With respect to the individual knowledge items, there appeared to be an understanding of the mode of transmission of SARS-CoV-2 across health worker categories. Possibly the most concerning of the knowledge items, was that almost half of the participants did not know that droplet and contact precautions were needed for both confirmed and suspected cases of COVID-19. Worryingly, this included almost 40% of nurses. Participants also struggled with the question related to hand hygiene with the majority of participants indicating alcohol-based hand rubs are always preferred over soap and water. This is not the case as the WHO recommends soap and water if hands are visibly soiled [12]. Contrary to the WHO’s guidance, an overwhelming majority of participants indicated boots, coveralls and aprons were needed in the routine care of COVID-19 patients. The WHO recommends a medical mask, eye protection, non-sterile long-sleeved gown and gloves [12]. A majority of participants indicated that suspected COVID-19 cases should be given N95 respirators to prevent transmission to health workers while the WHO recommends they be given medical masks [12].

Though the survey was conducted during the early stages of the epidemic in South Africa, the large proportion of participants who felt they were not adequately prepared to deal with COVID-19 was concerning. On a positive note, the health workers displaying attitudes and perceptions that would put themselves or others at risk were in the minority. Approximately 12% of health workers indicated they would choose not to wear the required PPE if it were uncomfortable. This is higher than a healthcare worker KAP survey on healthcare-associated tuberculosis where 6.2% of participants indicated they would not wear a mask if it were uncomfortable [21]. Approximately 9% of the participants would treat COVID-19 patients even if the required PPE were not available indicating the vast majority of participants understood the risk of treating COVID-19 patients without PPE. It is noteworthy that 10% of participants indicated they would still continue to report for duty even if they had symptoms suggestive of COVID-19 thereby putting colleagues and patients at risk. This may indicate a failure to effectively communicate the appropriate protocols to all health workers. While the proportion of participants indicating they would resign to avoid contact with COVID-19 patients was small (6.4%), it is nonetheless significant in a country already struggling with health workforce shortages in government facilities.

As health worker and health facility practices are intertwined, we asked our participants about both. Appropriate training has been shown to improve both infection prevention knowledge and practices [18] but only just over half of our participants had undergone infection prevention and control training. Given the important role that PPE plays in the prevention of transmission of SARS-CoV-2, it was concerning that more than 40% of participants had not received training on the correct use of PPE. The availability of infection prevention guidelines has also been associated with improved infection prevention knowledge and practices [18] and more than 70% of participants indicated these were available in their departments. It was concerning that not all participants had access to medical masks and gloves in their departments given that these should be routinely available. While the case numbers were low in South Africa at the time of the survey, these were expected to increase and lack of access to PPE would put these health workers at risk. Given the potential for SARS-CoV-2 to be transmitted by direct and indirect contact, the results for hand hygiene practices were encouraging. Our results differed markedly from the much lower 47% hand hygiene compliance rate after patient contact reported by Erasmus et al. [22].

### Limitations

Our survey was conducted as part of a rapid appraisal of COVID-19 occupational health and safety preparedness in four provinces of South Africa. We used convenience sampling to maximise the number of facilities that could be assessed in a short period of time. While we believe that our sample provides a fair reflection of the knowledge, attitudes and practices in health facilities in the four provinces, the non-probability sampling strategy needs to be borne in mind with respect to generalisation of the findings. Our study focussed on health workers that were likely to be the initial point of contact with COVID-19 cases at the various facilities. It is possible that their knowledge, attitudes, and practices differ from health workers working in wards and intensive care units who need to provide longer term care to COVID-19 patients. As we did not have access to patient statistics at facility level, we did not compare knowledge, attitudes and practices between health workers at healthcare facilities with high COVID-19 patient volumes and those at facilities who see few COVID-19 patients. The scientific community’s understanding of COVID-19 has been rapidly evolving since the first cases were discovered. We developed our questions on the accepted understanding of COVID-19 at the time of the survey and analysed the results as such. With respect to an acceptable knowledge score, it is not clear-cut as to what an appropriate cut-off would be particularly with clinical and non-clinical cadres involved in our study. We, therefore, presented results for an adequate score of 60% and a good score of 80%. Health worker reporting of practices may not reflect actual practices and it is likely that the self-reported hand hygiene compliance is an overestimate. Substantial discrepancies between self-reported hand hygiene practices and direct observation have been reported in the literature [23, 24].

## Conclusions

The majority of participants in our study demonstrated adequate knowledge of COVID-19 infection prevention and control with approximately a quarter of participants having good knowledge. A good knowledge score was associated with occupational category, age, and level of hospital. The attitudes of participants reflected a willingness to engage in appropriate SARS-CoV-2 infection prevention and control practices as well as a commitment to be involved in COVID-19 patient care. Ensuring adequate infection prevention and control training for all staff and universal access to appropriate PPE were identified as key areas that needed to be addressed. Interim and final reports were provided to the relevant provincial departments of health which identified key shortcomings that needed to be addressed.

## Data Availability

The datasets used and analysed during the current study are available from the corresponding author on reasonable request.

## Abbreviations

COVID-19: Coronavirus disease 19
SARS-CoV-2: severe acute respiratory syndrome coronavirus 2
WHO: World Health Organization
